# A Cell Atlas for the Mouse Brain

**DOI:** 10.3389/fninf.2018.00084

**Published:** 2018-11-28

**Authors:** Csaba Erö, Marc-Oliver Gewaltig, Daniel Keller, Henry Markram

**Affiliations:** Blue Brain Project, École Polytechnique Fédérale de Lausanne (EPFL), Lausanne, Switzerland

**Keywords:** cell numbers, neurons, glia, mouse brain, Allen brain atlas

## Abstract

Despite vast numbers of studies of stained cells in the mouse brain, no current brain atlas provides region-by-region neuron counts. In fact, neuron numbers are only available for about 4% of brain of regions and estimates often vary by as much as 3-fold. Here we provide a first 3D cell atlas for the whole mouse brain, showing cell positions constructed algorithmically from whole brain Nissl and gene expression stains, and compared against values from the literature. The atlas provides the densities and positions of all excitatory and inhibitory neurons, astrocytes, oligodendrocytes, and microglia in each of the 737 brain regions defined in the AMBA. The atlas is dynamic, allowing comparison with previously reported numbers, addition of cell types, and improvement of estimates as new data is integrated. The atlas also provides insights into cellular organization only possible at this whole brain scale, and is publicly available.

## Introduction

**Figure 1:**
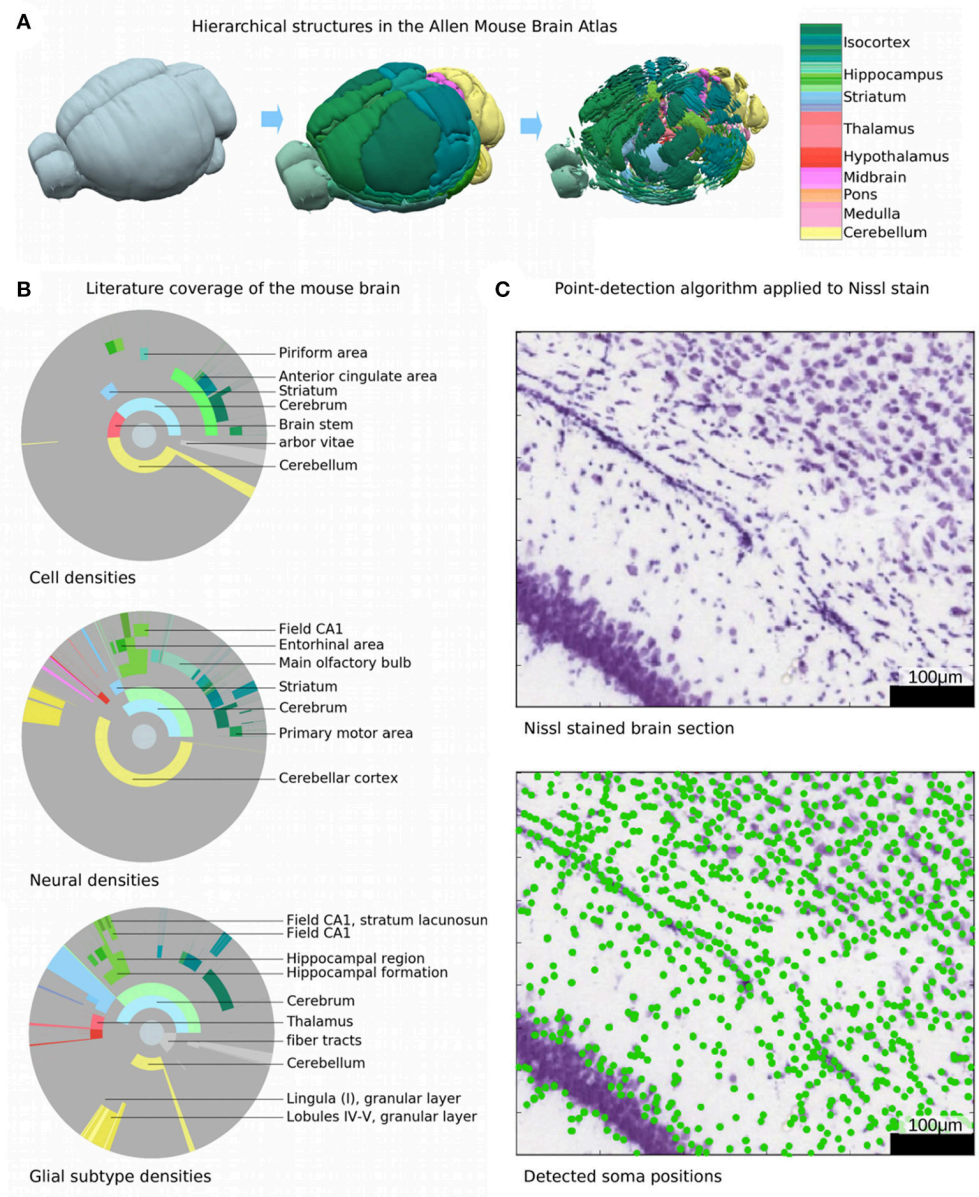
Current knowledge and decomposition of the mouse brain. **(A)** Illustration of the hierarchical definition of non-overlapping structures in the AMBA (Dong, 2008). The highest level comprises the entire brain, while the next level defines large brain structures such as olfactory bulb, cortex, or cerebellum. The next levels define progressively finer sub-structures. The color encodes the brain regions according to the AMBA, e.g., cortical areas are shown in green and cerebellar regions in yellow. **(B)** Illustration of the published information on densities or absolute numbers of cells, neurons, and glia in different regions of the mouse brain. Each disk is divided into rings and sectors. Each ring represents a hierarchical level in the AMBA and sectors represent the contained brain structures. The center of each disk represents the entire brain, each surrounding ring then represents the next hierarchy level. Colored areas represent regions where at least one study reports absolute numbers or densities, with color coded as in **(A)**. Gray areas represent brain regions where no literature data is available. The following disks from top to bottom illustrate for which regions numbers or cell densities have been published for cells, neurons, and glial subtypes, respectively. Most information is available for cortical and cerebellar regions, while much less is known about subcortical regions. **(C)** Illustration of automatically counting cell soma from Nissl stains. Top, original Nissl stain from AIBS, with soma stained in blue. Bottom: overlaid cell positions as detected with a state-of-the-art detection algorithm. The algorithm performs well in areas where cell soma are well-separated. In areas where cells are so dense that cell soma overlap, automatic cell counting fails.

Since the seminal work of Ramon y Cajal over a century ago, a vast number of studies have used a wide range of methods to count stained cells in the brain. Despite these efforts, neuron counts cover only about 4% of the hierarchical regions defined in the Allen Brain Atlas (Lein et al., 2007; Dong, 2008). Counts of glia, or the ratio of glia to neurons, are even less common and counts of neurons and glia belonging to specific types are still to be established (Figure 1B). One reason for this paucity of data is that most cell counting studies focus on large regions without examining their subdivisions (Figure 1B). For example, while it is possible to find reported cell and neuron numbers for larger cortical regions such as visual, somatosensory, or auditory areas (Herculano-Houzel et al., 2013), the cellular content of their subdivisions into smaller functional areas or even layers remains unknown. Another reason is that some brain structures such as the barrel cortex or the hippocampus have been studied extensively, while other regions such as the pontine nuclei or anterior olfactory nuclei are studied less frequently or not at all.

Considering all available reports on cell densities in the mouse brain, it does not seem possible to reach ground-truth values because no estimates have been reliably reproduced (Keller et al., 2018). In fact, cell counts from any two of studies of the same region vary by a median of 1.8-fold and a mean of 4.1-fold, with some estimates varying as much as 13.1-fold. Herculano-Houzel et al. (2013) for instance, report a mean neuron density in the frontal cortex at 6.68·10^4^ mm^−3^, while Schmid et al. (2013) obtain 12.3·10^4^ mm^−3^. While recent studies have stained and counted cells with much greater precision than ever before, our state of knowledge today remains a rather rough notion of the number of cells, neurons and glia in the whole mouse brain and in some of the large brain regions. Reliable estimates would provide a solid foundation for large initiatives to understand the brain (Markram et al., 2011) to reach a consensus on cell types (Jorgenson et al., 2015), and to reconstruct and simulate the brain (Markram, 2006).

Agreement on the total number neurons in the whole mouse brain has only recently emerged: around 70 million (Herculano-Houzel et al., 2006). Confidence in this estimate has not come as much from reproducible estimates, but from the ability to count all the neuronal nuclei present in homogenized brain tissue from the whole brain—a method that eliminates errors in manual counting of stained cells from small samples (Herculano-Houzel and Lent, 2005). It has however been difficult to extend this approach to specific brain regions and to smaller sub-regions, areas, modules, layers, and nuclei requiring precise excision before homogenization, still leaving vast gaps in our knowledge of cell numbers in the different regions of the brain. New microscopic techniques can visualize labeled neurons in all areas of the brain, and have resulted in the CUBIC-X (Murakami et al., 2018) and qBrain (Kim et al., 2017) atlases. As new information of this type becomes available, the need to integrate different data sets becomes more pressing.

Another invaluable dataset is the mouse whole-brain atlas made available by the Allen Institute for Brain Science (AIBS) (Lein et al., 2007; Dong, 2008). This atlas contains Nissl stained microscopy slices for the whole brain, as well as most genes used *in situ* hybridization studies. In principle, the Nissl stained whole brain atlas contains all the data needed to estimate the number of cells in the whole mouse brain, and in each brain region—if the cells could be counted reliably. The nearly 20,000 whole brain gene expression atlases also, in principle, contain information that could help estimate the number for different cell-types such as neurons and glia, and even further subdivide cells into excitatory and inhibitory neurons, and astrocytes, oligodendrocytes, and microglia.

The problem is that even assuming perfect staining, manual counting of all these cells would not only be an enormously laborious task, but more importantly would be prone to counting errors, missed cells, duplicate cell counts and error expansions when extrapolating local cell density estimates to a large region or to the whole brain. Deviations in large regions can be significant, as the error obtained in a small volume grows alongside the cell counts when scaling up the volume. Errors can also increase in smaller brain regions, sub-regions, areas or layers (Figure 1A) because they are less reliably or reproducibly isolated. Furthermore, even the enormous dataset obtained for the Allen Brain Atlas is not sufficient to obtain the full individual biological variability since the same value for any brain region would be required for many animals. Obtaining cell counts for all brain regions across different ages also awaits a faster and more reliable approach.

Point-detection algorithms could automatically count cells in stained tissue, but they systematically underestimate numbers because cells spatially overlap. This error grows as the cell density rises (Figure 1C). Even if the errors are only significant for a small portion of the brain volume where very high cell densities are found, they cannot be neglected because they would contain some of the largest cell numbers.

To overcome these challenges, we chose to build a dynamically generated cell atlas of the mouse brain that can integrate diverse datasets to converge toward ground-truth estimates, in principle for all cell-types in all brain regions. We used the 3D volume framework of the Allen Mouse Brain Atlas (AMBA) (Lein et al., 2007) to delineate all the brain regions, and filled the volume of each of the brain regions with cells according to data-driven and algorithmically generated estimates. Such estimates were obtained by loading whole brain staining data from the AMBA, aligning and voxelizing the slices, and filling each brain region with cells corresponding to the computed densities. We used a variety of whole brain image datasets, including Nissl-staining for cells and genetic marker stains to distinguish neurons from glia, and finally the main types of neurons (excitatory and inhibitory) and glia (astrocytes, oligodendrocytes, and microglia). We also used some values reported from anatomical experiments in the literature. Finally, we compared the estimates against values reported in the literature that were not used in the reconstruction of the cell densities. We also constructed the Atlas to enable further integration of data to facilitate convergence toward ground-truth, or at least toward a general consensus on cell numbers. Finally, for those brain regions where the further subdivisions of cell-types are known, the atlas allows for refining the composition of cells.

Multi-origin constraints are essential to overcome many of the difficulties of counting cells in large tissue volumes and allow reasonable estimation of the number of cells in every brain region. We can thus provide, for the first time, estimates of the numbers and densities of the main classes of neurons (excitatory and inhibitory) and glia (astrocytes, oligodendrocytes and microglia) for the entire mouse brain, including the smallest brain regions, sub-regions, nuclei, and layers. Placing all cells in the 3D volume of the brain and in brain regions also yields the spatial distribution of cells and in fact provides a 3D location for every cell. The cell atlas can become more precise as more data is integrated (e.g., high resolution stainings; new stainings, single cell transcriptomic data, etc.), and in the future estimates for the number of cell-types at finer levels of classification (morphology, electrical, molecular, etc.). Finally, the model shown in the cell atlas can be generated multiple times with a range of constraints to capture individual biological variability. The 3D cell atlas has been made publicly available as an online resource at bbp.epfl.ch/nexus/cell-atlas.

Some of the limitations of our approach for obtaining cellular distributions throughout the brain can be summarized as follows. We assumed the stained intensity of Nissl and other genetic markers to be a good indicator of soma density specific to the population of interest, without significantly staining axons and dendrites. Overall, we applied our density calibration uniformly in every brain region, assuming similar soma sizes within the three largest areas of the brain. We did not take in account spatial exclusion between somata allowing them to be located arbitrarily close to each other, as this effect was statistically insignificant over entire regions. Finally, all marker data were based on individual subjects. These assumptions will be described in detail in the following sections.

## Results

**Figure 2:**
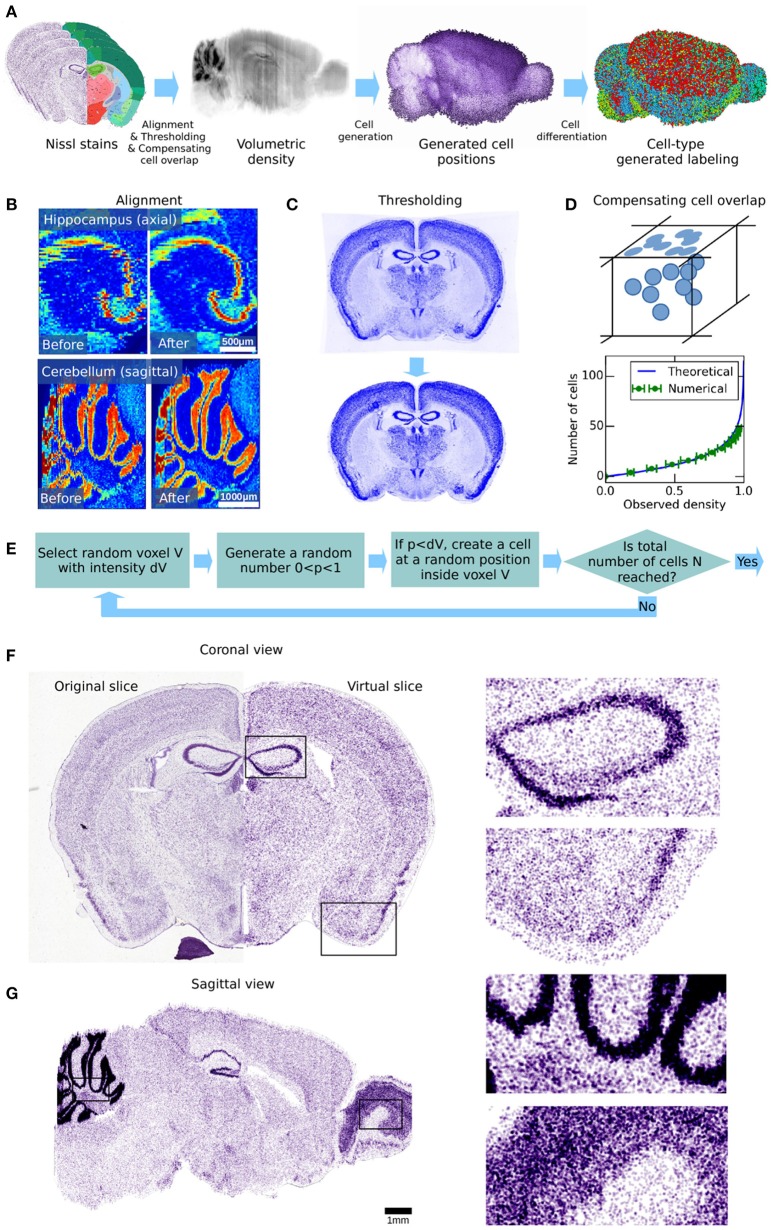
Workflow for generating cell positions for the whole mouse brain. **(A)** Illustration of the different processing steps of our workflow. The 1st panel shows the AIBS Nissl stained microscopy slices. These are processed to obtain a volumetric dataset of cell density throughout the brain, shown in the 2nd panel. The cell positions that are created from it using an acceptance-rejection algorithm are shown in the 3rd panel. Finally, the generated cells are differentiated by type, as shown in the 4th panel. The cell types shown are glial cells (green), excitatory neurons (blue) and inhibitory neurons (red). The different processing steps are illustrated in **(B–E)**. **(B)** Illustration of how the volumetric dataset can be improved by automatic non-rigid alignment. Two regions in the volumetric dataset are shown as example, before and after non-rigid realignment. An improvement in the cohesion of the brain structures can be clearly seen. **(C)** Coronal slice of the genetic marker Nrn1 before and after thresholding, showing the additional dye uniformly present even in brain regions that lack the genetically targeted cells. Thus, the average signal outside of the brain volume was subtracted from the total signal. While this procedure had little effect on the Nissl stained slices, it had more impact on the genetic markers that were used in the later stages of this paper. **(D)** Illustration of how cells overlap when observed on a 2-dimensional plane such as a microscopy image. This effect was corrected by a mathematical function which was applied to approximate the actual number of cells as a function of the observed voxel intensity. This function was numerically validated, and then applied to the entire density dataset. **(E)** Acceptance-rejection algorithm used for the generation of cell positions. This is an iterative step, and was repeated until the targeted number of cells in the brain was reached. **(F)** Comparison between the original Nissl stained slice and its virtual counterpart, obtained using the cell positions generated in our workflow, in coronal view. Both show similar structures and correlate quite well despite the generated cells being displayed as simple spheres of uniform size. **(G)** Virtual slice obtained using the cell positions generated in our workflow, in sagittal view.

Our 3D cell atlas is developed in three steps (Figure 2A): In the first step, we start with the Nissl microscopy dataset from the AMBA. After re-aligning, thresholding and compensating for cell-overlap, we obtain a volumetric estimate of the cell density. In the second step, we use the volumetric cell density to generate cell positions in 3D space, delineated by the AMBA. Finally, we use various genetic marker staining data as well as literature data to label the generated cells as either excitatory and inhibitory neurons as well as subtypes of glia (astrocytes, oligodendrocytes, and microglia).

### Estimating the volumetric cell density

The gray-scale Nissl volume and the structural annotation volume of the AMBA (Lein et al., 2007; Dong, 2008) are based on 509 Nissl stained coronal sections of 25 μm thickness. The structural annotation volume identifies 737 brain structures. In the Nissl volume (v. 2011), individual coronal slices of the original image stack are still visible (Figure 2B, left). In order to estimate the cell densities also in the smallest brain structures, we re-aligned adjacent slices of the Nissl volume by applying an automated non-rigid alignment algorithm, based on Kroon (2008), described in Appendix section 1.1 in Supplementary Material. This not only improved the offset between adjacent slices, but also partially corrected the spatial warping in the images created during image acquisition (Figure 2B, right). The result is a realigned 3D atlas with the same voxel resolution as the original reference atlas, but with improved alignment between coronal slices. This improved reference atlas was used in all subsequent stages of our analysis.

**Figure 3:**
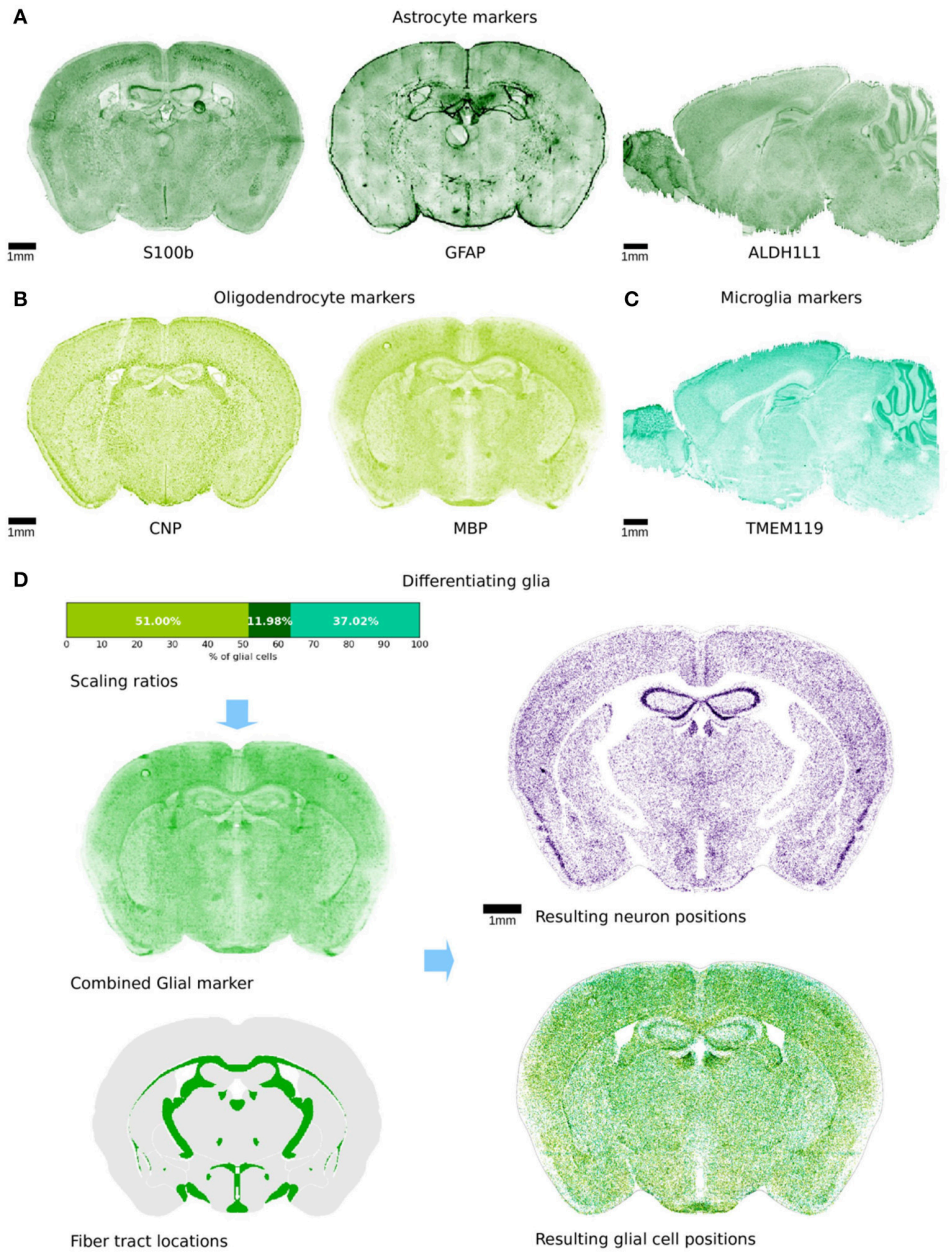
Differentiation of glial cells. **(A–C)** Illustration of the variety of genetic markers that were used to obtain an approximation of glia density. The colors represent astrocyte markers **(A)**, oligodendrocyte markers **(B)**, and microglia markers **(C)**. Some of the marker experiments were only available in sagittal view and are therefore shown in that arrangement. All markers exhibit a fairly high resolution, as the microscopy slices were manually realigned using a non-rigid landmark based alignment. Some imaging artifacts are visible and cannot be corrected. **(D)** Top left: the 3 markers were combined with the illustrated ratios. The resulting volumetric dataset was used as an approximation for glia density throughout the brain volume. Bottom left: the regions shown in green are the AIBS annotated fiber tracts that are known to only contain non-neuronal cells. Top right: remaining neuron positions in a virtual slice after the differentiation procedure. Bottom right: glia cells differentiated during the procedure. Colors are as in **(A–C)**.

**Figure 4:**
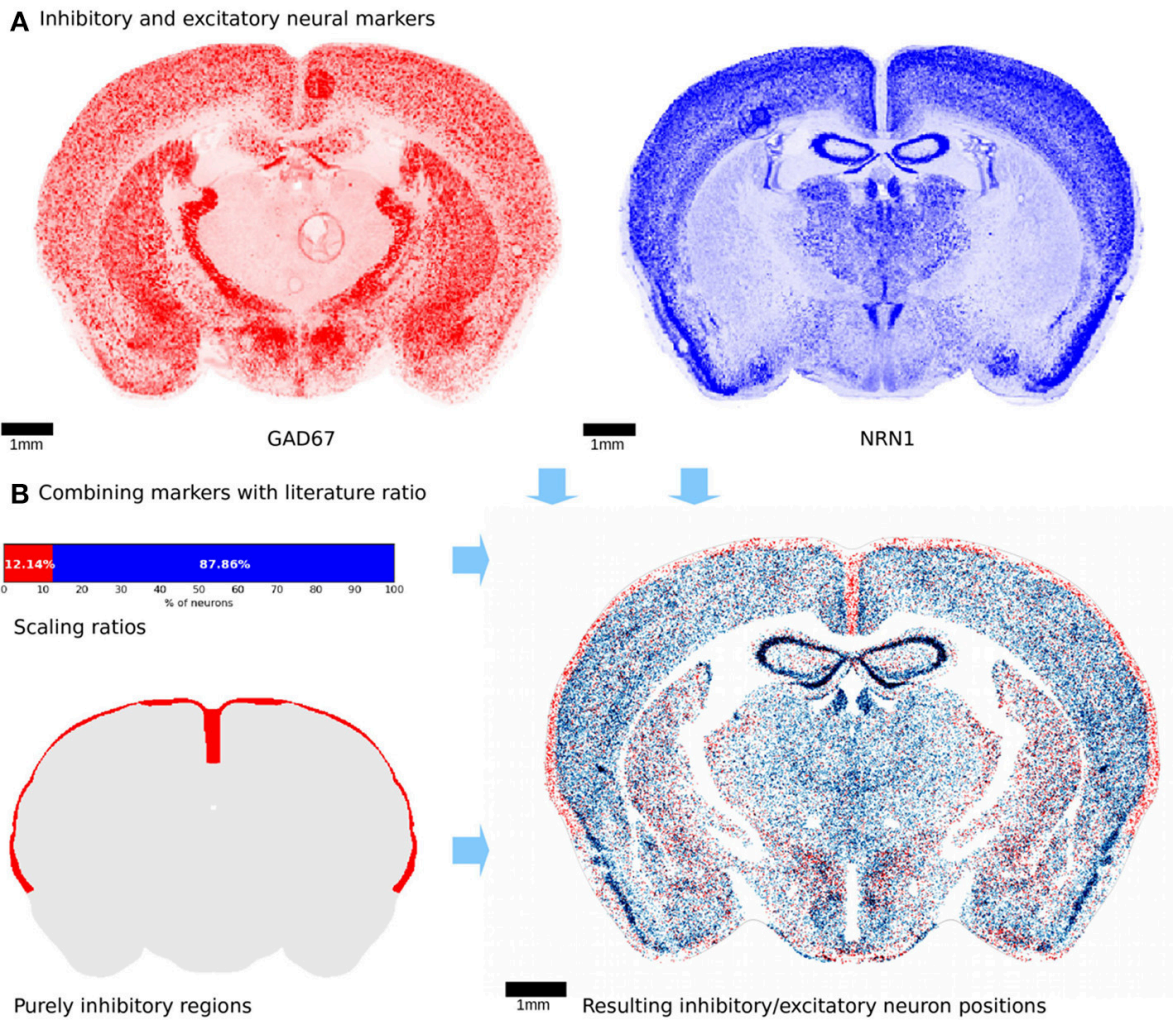
Neuron differentiation into excitatory and inhibitory types. **(A)** Illustration of genetic marker experiments used to distinguish inhibitory from excitatory neurons. The inhibitory marker is shown in red while the excitatory marker is blue. The two markers exhibit clear differences in terms of density, especially in the thalamic regions. Both marker experiments were realigned manually using landmark based non-rigid alignment. Some imaging artifacts are visible and cannot be corrected. **(B)** Top left: the 2 markers were combined with the illustrated ratios from literature. Bottom left: regions shown in red that are known to contain only inhibitory neurons. This additional constraint was applied to the differentiation procedure. Right: virtual slice showing positions of inhibitory and excitatory neurons. Colors are as in **(A)**. Similarly, to the markers, the same regions seem to exhibit a fairly high density of inhibitory neurons, while others are mostly excitatory.

For the genetic marker stains (Lein et al., 2007) the resolution of the volumetric data was too low for our purpose (200 μm). We therefore had to realign the original microscopy images of all used markers, with a manual landmark-based non-rigid alignment method (Kroon, 2008). Using around 40 fiducial points per image allowed us to create new voxel datasets with an effective resolution of 25 μm (Figures 3A–C, **4A**). As opposed to traditional affine realignment methods, the non-rigid deformation that we applied was able to warp images in space using an overlay grid, and therefore had a much greater degree of freedom. For an example video of the warping effect resulting of the non-linear alignment, see Supplementary Materials. To remove the constant baseline signal and to enhance the contrast of the images, we further applied a simple threshold function (Figure 2C).

From the volumetric data-sets, we then estimated the volumetric cell density by applying a transfer function that compensates the possible cell overlap in the 25 μm slices (Figure 2D), which can be written as

D = f(V) = −ln(1−V) · A

Where D is the final relative cell density, V is the observed density, and A is a constant. This function was obtained by calculating how many actual cells are needed in a volume to result in a given observed density on the microscopy image.

The details of the automatic and manual alignment methods as well as the derivation of the transfer function are described in Appendix section 1.2 in Supplementary Material.

### Generating cell positions

To generate the cell positions for the whole mouse brain, we used the following Monte-Carlo algorithm (Figure 2E): First, the volumetric density dataset was normalized to values between zero and one. The algorithm then picked a random voxel from the volume and a uniformly random number x between 0 and 1. If the density at the voxel was larger than x, a cell was registered at a random position within the voxel. This procedure was repeated until the desired number of cells *N* was reached.

Our first approach was to stop placing cells when the total number reported for the whole brain (*N* = 111,080,000) by Herculano-Houzel et al. (2011) was reached. However, this resulted in unrealistic values for the predicted cell densities, particularly for the cerebellum where the extremely large number of tiny granule cells is much higher than the Nissl stain could suggest. We remedied this problem by constraining the cell numbers at three different areas: in the cerebellum (*N*_*Cerebellum*_ = 49,170,000), in the isocortex (*N*_*Isocortex*_ = 23,378,142), and the rest of the brain (*N*_*RoB*_ = 38,531,858), as reported also by Herculano-Houzel et al. (2011).

With these constraints, we estimated the positions of cells in the entire brain. Figure 2F shows a comparison between an original Nissl stained image from the AMBA and a reconstructed slice from our cell atlas, in coronal view. Overall the reconstructed slice matched the original well, as most structures can be recognized in both slices, despite the generated cells being displayed as simple spheres of uniform size. The main noticeable difference between the original and the artificial slice is a slight loss of spatial resolution, due to the low-pass filtering during the density estimation. As a result, cells in the model are less accurately distributed than in the original slice and the boundaries of smaller structures, although present, are less well-defined. While the original Nissl stained images are only available in coronal view, our cell atlas allows a sagittal view (Figure 2G), revealing slight traces of coronal misalignment, despite the realignment of the dataset. It also has to be noted that the cell generation algorithm does not take in account spatial exclusion between somata, as it does not seem to have any effects on the statistical distribution of cells at the level of entire brain regions, and also because the sizes of individual cells are not known.

### Genetic markers

Although the positions of all cells in the brain are now defined, their type remains unknown. We therefore decided to use the genome-wide atlas of gene expression produced by the AIBS, registered to the same reference atlas to further differentiate the cells into subtypes of glia cells such as astrocytes, oligodendrocytes, and microglia, and inhibitory or excitatory neurons, neglecting further subdivisions for the moment. Although both excitatory and inhibitory neurons can be divided into further sub-types, differentiating them in all brain regions would require a complex combination of many additional markers.

### Glia differentiation

In order to distinguish glia from neurons, we needed to know the volumetric density of either neurons or glia. In this case, we used commonly-accepted glial genetic markers to identify glia: astrocytes GFAP (Jacque et al., 1978) and S100b (Hachem et al., 2005) and ALDH1L1 (Cahoy et al., 2008), oligodendrocytes MBP (Hokama et al., 1986), CNP (Gravel et al., 1996), and microglia TMEM119 (Satoh et al., 2016) markers (Figures 3A–C). Unfortunately, the markers are not specific to all cells of any type, and images for any one marker were furthermore noisy. Therefore, an estimate based on a single marker would be expected to underestimate the overall density of glia and overestimate some subtypes of cells where the gene is also expressed in multiple glial cell types. We thus used multiple markers in conjunction with transcriptome data (Zeisel et al., 2015) to estimate the proportion of GFAP and S100b and ALDH1L1 relative to the entirety of all astrocytes, and obtained an estimate for glia cell density according to the following equation:

$$\begin{array}{l} {\text{D}}_{\text{GLIA}}\;={\text{ S}}_{\text{oligodendroctes}}\cdot ({\text{C}}_{\text{CNP}}\;\cdot \text{ CNP}+{\text{C}}_{\text{MBP}}\;\cdot \text{ Mbp})\\ \;+\;{\text{S}}_{\text{Microglia}}\;\cdot \text{ TMEM119}+{\text{S}}_{\text{Astrocytes}}\;\cdot \;(\text{CGFAP }\cdot \text{ GFAP}\\ \;+\text{ CS100b }\cdot \text{ s100b }+\;{\text{C}}_{\text{ALDH1L1}}\;\cdot \text{ALDHL1L1}) \end{array}$$

CNP, MBP, GFAP, S100b, ALDH1L1, and TMEM119 are the observed image intensities for the respective markers. The weight factors *C*_*marker*_ for the genetic markers of a given cell type are given by

$${\text{C}}_{\text{marker}}=\frac{{\text{1/E}}_{\text{marker}}}{{\text{N}}_{\text{Markers}}}$$

where E_marker_ is the expression intensity of the marker, and N_markers_ is the number of markers specific to that cell type. We computed the global scaling factors S_celltype_ in order to best match known density values for several regions (Table 1).

**Table 1:** Average glia density (mm^−3^) in different brain regions, as found in several literature sources (Keller et al., 2018).

**Region**	***D_Oligodendrocytes_***	***D_Astrocytes_***	***D_Microglia_***
Cerebellum	15,000 (Förster, 2008) 12,500 (San Jose et al., 2001) 1840.8 CC+ (Relucio, 2011) **Mean and STD: 13,750** ± **1,768**	**1,512** (Rockland and Defelipe, 2011)	9,090 (Rockland and Defelipe, 2011) 8,158 (Journiac et al., 2005) **Mean and STD : 8,624** ± **659**
Cortex	**12,500** (Rockland and Defelipe, 2011)	**15,696** (Grosche et al., 2013)	**6,500** (Nimmerjahn et al., 2005) (Lawson et al., 1990)
Hippocampus	**9,425** (Geisert et al., 2002)	29,008 (Shimada et al., 1992) 20,904 (Grosche et al., 2013) 12,226 (Schmalbach et al., 2015) 4,811 (Geisert et al., 2002) **Mean and STD: 16,737** ± **10,496**	4,353 (Geisert et al., 2002) 2,143 (Schmalbach et al., 2015) **Mean and STD: 3,248** ± **1,563**
Striatum	12,000 (Steen, 2006) 5,300 (Förster, 2008) 12,550 (Binder et al., 2011) **Mean and STD: 9,950** ± **4,036**	4,002 (San Jose et al., 2001) 9,000 (Steen, 2006) 4,400 (Globus pallidus) (Charron et al., 2014) 12,000 Dorsal caudate/putamen (Schmid et al., 2013) 19,000 Medial caudate/putamen (Schmid et al., 2013) 10800 (Förster, 2008) **Mean and STD: 9,867** ± **5,547**	12,403 (San Jose et al., 2001) 15,000 (Steen, 2006) 9,700 (Lawson et al., 1990) 12,100 globus pallidus (Lawson et al., 1990) 11,300 (Förster, 2008) **Mean and STD: 12,101** ± **1,930**

*Bold values represent the final values used for each region*.

Finally, we applied the previously defined voxel density function *D* = *f(V)*, to the voxel intensity dataset to approximate spatial glia density.

As an initial constraint, all cells present in the annotated fiber tract regions were classified as glia only. For all remaining regions, we distinguished cells into glia and neurons according to their spatial density obtained from the glia marker dataset, and using an acceptance-rejection method similar to the one used for the cell creation step. To do this, the volumetric glia density dataset was first normalized to values between zero and one. The algorithm then picked a random voxel from the volume and a uniformly random number x between 0 and 1. If the density at the voxel was larger than *x*, a random cell in that voxel was labeled as glia. This procedure was repeated until we obtained a total glia cell/neuron ratio for the whole brain of *rglia* = 35.4% (Herculano-Houzel et al., 2011). The resulting glia positions could then be separated from the remaining neuron positions (Figure 3D). The annotated fiber tracts did not contain any neurons, due to the imposed constraints.

### Neuron type differentiation

We wanted to further subdivide neurons into inhibitory and excitatory types. A variety of markers that stain inhibitory and excitatory neurons exist. GAD67 is mainly expressed in inhibitory neurons, and can thus be used to estimate their density, while NRN1 is mainly expressed in excitatory neurons (Figure 4A). We normalized the GAD67 marker with a sum of both markers, with an overall ratio of 7.94% between inhibitory neurons (Kim et al., 2017). We then used the resulting volumetric inhibitory marker density.

Before applying the acceptance-rejection algorithm, certain regions with a known highly inhibitory neuronal content, were constrained. These were all layer 1 cortical regions, and could be distinguished using the AMBA annotation dataset. Similar to the glia differentiation step, existing neurons were iteratively assigned as being inhibitory following the density of the inhibitory volumetric dataset, until the total percentage of inhibitory neurons reached 7.94%. The resulting neuron type positions (Figure 4B) show a distribution that follows closely the spatial density provided by the markers.

### Modulatory neurons

We investigated the distribution of three main modulatory neuron types in the brain: dopaminergic, serotonergic, and acetylcholinergic. As the first two were known to be more localized in specific areas of the brain, we manually assigned them to their corresponding annotated regions. We found the Substantia Nigra (SN) and Ventral Tegmental Area (VTA) of the Midbrain to be composed to 37.6 and 63.8% of dopaminergic neurons (Nair-Roberts et al., 2008), which we applied as a spatially uniform constraint throughout these regions. We further found the Raphe Nuclei (RN) of the Midbrain to contain 9,000 serotonergic neurons (Mlinar et al., 2016), which using our generated neural numbers yielded a fraction of 60.2% in that region. Finally, as acetylcholinergic neurons were known to be more spread out throughout the entire brain volume, we used the relative volumetric density of the ACh marker from the AIBS to approximate their distribution. As we had no global constraint on the total number of acetylcholinergic neurons in the brain, we used a region-specific constraint of 0.75% for the Striatum (Tepper et al., 2010) to normalize the relative volumetric density. Due to the region-specific localization of most of our neuromodulatory cells as well as their low counts, we did not examine their statistical distribution throughout the brain volume.

### Whole-brain composition

**Figure 5:**
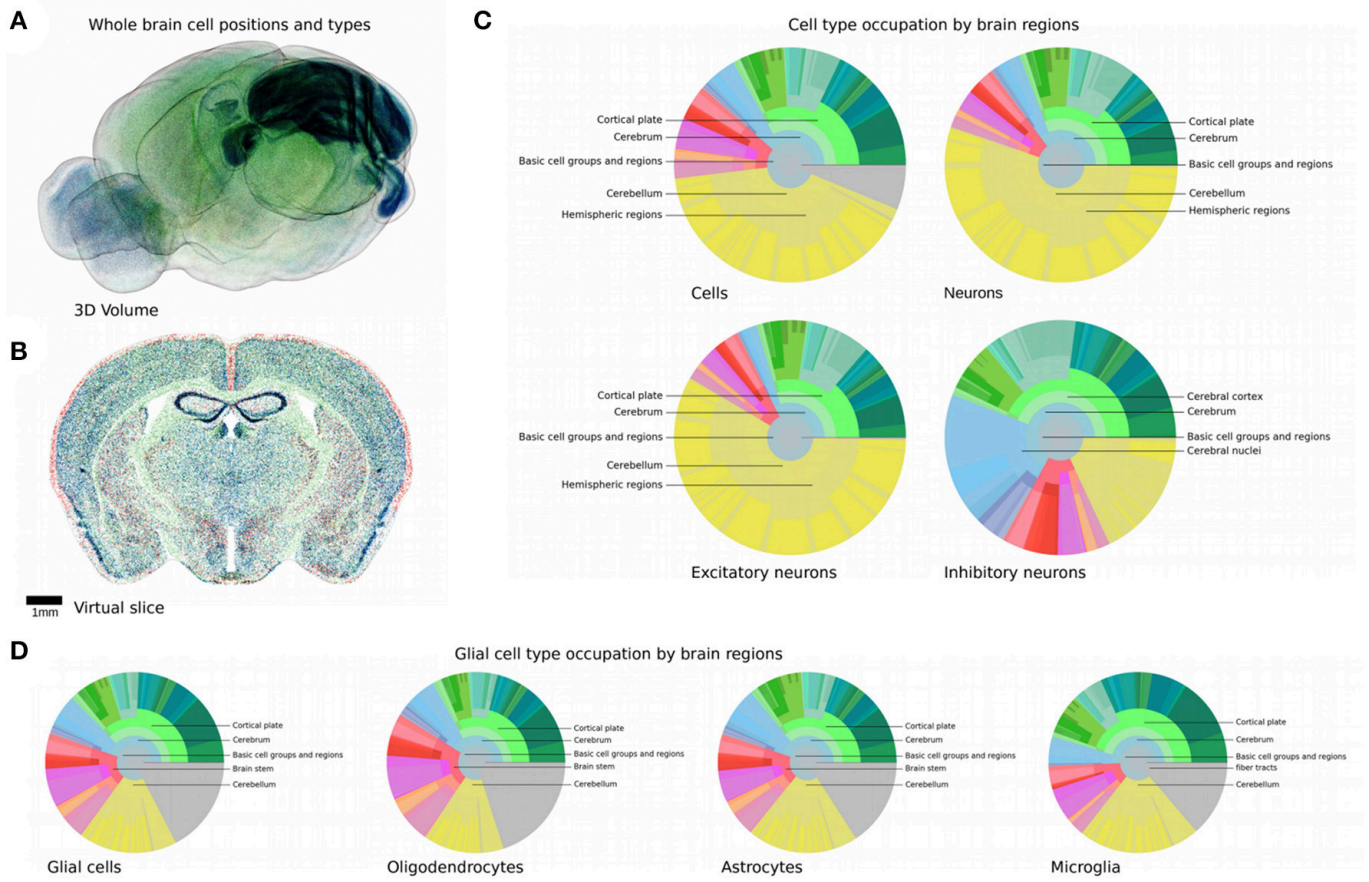
Reconstructed cell positions and types in the mouse brain. **(A,B)** Global overview of positions and types of all generated cells. **(A)** Labeled cells in the full 3D volume with outside boundary of the brain shown for clarity. **(B)** *In silico* coronal slice of 25 μm thickness. Glial cells are shown in green, inhibitory and excitatory neurons are shown red and blue, respectively. The cerebellum and the hippocampus are clearly visible due to their high cell densities and their distinctive shapes. **(C,D)** Composition of all regions of the mouse brain, in terms of cells, neurons and glia. Display and colors as in Figure 1B but with the estimates generated by our workflow. Gray areas represent fiber tracts.

The result of our algorithm is a reconstruction of all cells in the mouse brain, where each cell has a location and is assigned a type. All cells can be visualized in their positions in the brain volume (Figure 5A). A very high density is observed in the cerebellar region, as expected. Furthermore, the olfactory bulb mainly contains excitatory neurons, which is most likely because most coronal markers were missing coronal slices in that area and thus had to be extrapolated from the next closest slice, leaving this region with a higher uncertainty than the rest of the brain. A better overview of the generated brain structures can be obtained by visualizing all cells in an *in-silico* coronal slice (Figure 5B). This view is a combination of the previously visualized glial cells and neurons (Figures 3B, **4B**) and shows the cell-type specific distribution of different regions such as the inhibitory-only first cortical layers. In addition, the thalamus seems to be strongly polarized into either mostly inhibitory or mostly excitatory areas.

The composition of the entire brain can be read out and analyzed for both glia cells and neuron types and numbers. Figures 5C,D summarizes the results in a similar fashion as Figure 1B, but with all regions of the brain filled. This is a considerable improvement compared to the coverage presented earlier (Figure 1B), which barely filled 4% of the pie chart representing the regional hierarchy of the brain, despite an extensive literature study. The complete list of the generated cell, glia, and neuron densities and numbers can be found in Supplementary Materials.

The most striking feature is the size of the area occupied by the cerebellum in terms of cell numbers, compared to the rest of the brain. 42.0% of all cells are located there, and 59.8% of all excitatory neurons (Figures 5C,D). This is especially remarkable as this region only accounts for about 10% of the brain volume. This is because of the very small size of granule cells that make up this region, allowing them to be packed with an extremely high density. Additionally, 96% of the cerebellum is excitatory, with 37.7 million excitatory against 1.1 million inhibitory neurons, the latter however representing 18.9% of all inhibitory neurons in the brain. The region with the second highest cell number after the cerebellum is the cortical plate, mostly due to its large volume. Over 50% of the inhibitory neurons of the whole brain are located there. The striatum seems to be predominantly inhibitory, which is consistent with experimental observations (Tepper et al., 2010).

Glial cells seem to follow a more uniform distribution than neuron types, with their main feature being their presence in fiber tracts as well. While glia subtypes only differ slightly in their regional distributions, oligodendrocytes seem to be more prevalent in fiber tracts than the other two, which can be explained by their role in maintaining myelin sheets around long-range fibers (Figure 5D).

### Cell type correlations

**Figure 6:**
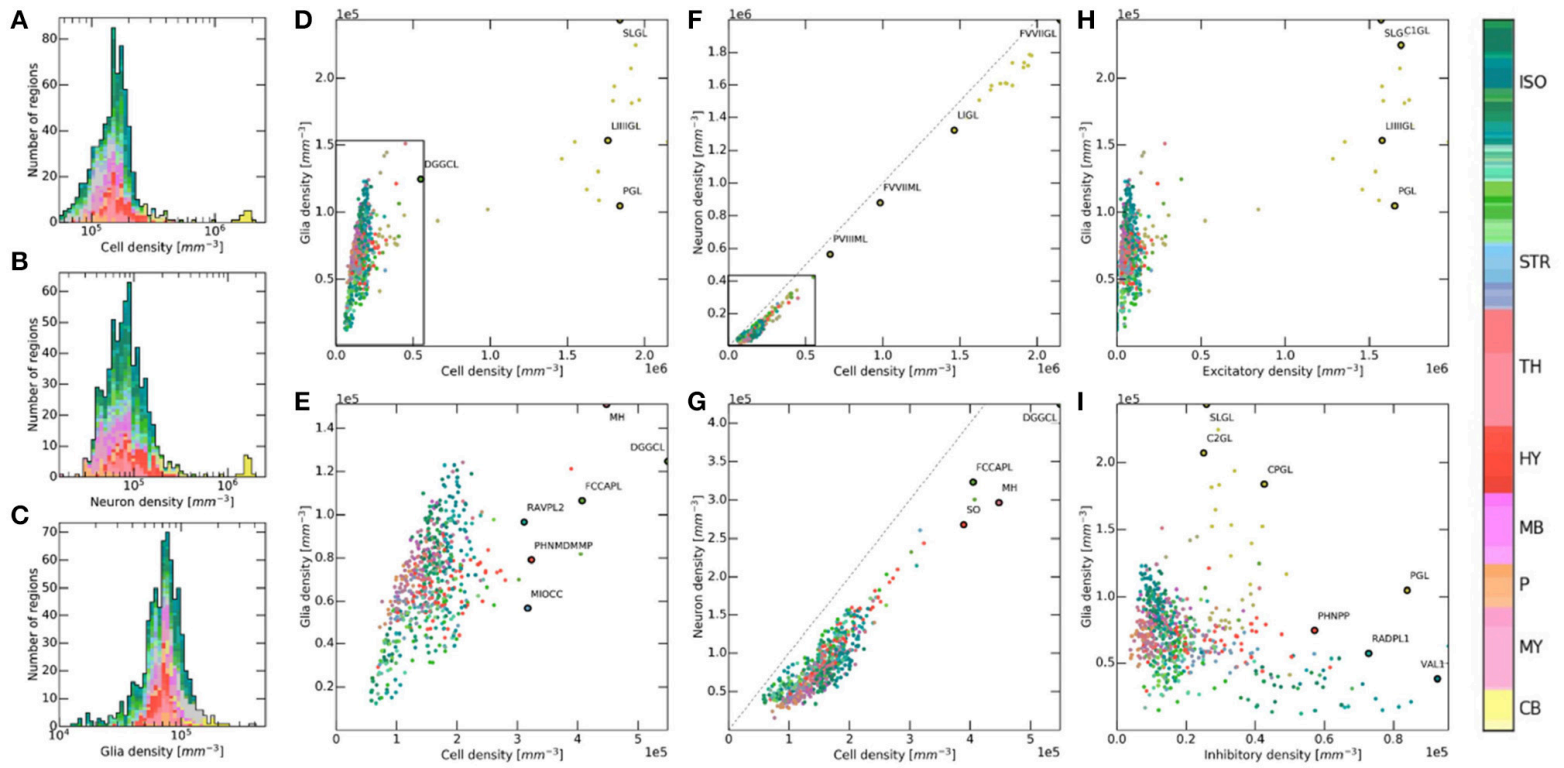
Relation between cell, glia, and neuron densities across brain regions. Color codes the brain region according to the AMBA notation (colorbar to the right). **(A–C)** Histogram of brain regions in terms of their density for cells, neurons and glia. Each region is shown with the same size, on a logarithmic density axis. **(D,E)** Inter-dependence between glia and cell densities, shown with (top) and without (bottom) the cerebellum. The granule layers of the cerebellum become isolated due to their extremely high density. Hierarchically close regions often tend to form clusters, even though no regional distinction was made during the workflow. DGGCL, Dentate gyrus, granule cell layer; PGL, Paraflocculus, granular layer; SLGL, Simple lobule, granular layer; LIIIIGL, Lobule III, granular layer; RAVPL2, Retrosplenial area, ventral part, layer 2; MH, Medial habenula; FCCAPL, Field CA3, pyramidal layer; PHNMDMMP, Paraventricular hypothalamic nucleus, magnocellular division, medial magnocellular part; MIOCC, Major island of Calleja. **(F,G)** Inter-dependence between neuron and cell densities, shown with (top) and without (bottom) the cerebellum. The dashed line represents equal densities, where cells would be comprised of only neurons. FVVIIML, Folium-tuber vermis VII, molecular layer; FVVIIGL, Folium-tuber vermis VII, granular layer; LIGL, Lingula I, granular layer; PVIIIML, Pyramus VIII, molecular layer; FCCAPL, Field CA2, pyramidal layer; SO, Subfornical organ; DGGCL, Dentate gyrus, granule cell layer; MH, Medial habenula. **(H,I)** Inter-dependence between glia and neuron densities, shown for excitatory neurons (top) and inhibitory neurons (bottom). C1GL, Crus 1, granular layer; PGL, Paraflocculus, granular layer; SLGL, Simple lobule, granular layer; LIIIIGL, Lobule III, granular layer; VAL1, posteromedial visual area, layer 1; C2GL, Crus 2, granular layer; CPGL, Copula pyramidis, granular layer; PHNPP, Periventricular hypothalamic nucleus, posterior part; RADPL1, Retrosplenial area, dorsal part, layer 1.

The histograms of brain regions in terms of their density for cells, neurons and glia (Figure 6A) show the cerebellar regions being responsible for the long-tail of the distributions. The latter is well visible despite the logarithmical scale of the density axis. Interestingly this is not the case for glia, which suggests that its density is not always proportional to that of cells or neurons. Furthermore, the glia and neuron distributions seem to also be diverging for low densities, with more regions having a lower glial than neuronal density. Overall, the distribution of glia seems to be narrower than that of cells and neurons, implying a more uniform distribution throughout the brain. Finally, one has to be careful with the interpretation of the exact shape of the histogram, as regions vary in terms of volume as well, with smaller regions appearing in the same size than larger ones.

Next we looked at possible correlations between different cell type densities throughout brain regions. We visualized each of the latter as separate dots in density and cell-type ratio space, and compared them (Figure 6). Overall there seemed to be a noticeable level of clustering between members of the same larger regions. Indeed, dots of the same color tended to be close to each other, even though regional annotation data was never used besides for setting global cell number constraints. This suggests that structurally close regions in the brain also tend to exhibit similar density properties. This is of course not a strict rule as the regions plotted are never perfectly clustered to the point that they could be distinguished from each other by their density properties alone. As expected, cerebellar regions exhibit density values so high that they are usually located at the extremes of the density space, when not excluded from the figure.

A roughly linear relationship can be observed between glia and cells in the brain for low to medium densities (Figure 6D). This correlation was however not consistent enough to predict glia densities from cell densities alone, as the figure exhibits a cone-like shape rather than a linear relationship for low numbers (Figure 6E). This relationship further seems to break down for densities higher than 2.5·10^5^ mm^−3^, as glial densities cannot follow cell densities anymore. This is especially the case of granule layers of the cerebellum as they exhibit extremely high densities. Although glial cells are often assumed to follow this proportional relationship due to their role in both maintaining the network structure and providing neurons with metabolic support, this does not seem to always be the case here. Figures 6F,G shows a similar effect but from a different point of view. Indeed, neuronal density seems to follow cellular density linearly for low values, while it seems to follow a constant offset for higher densities. This suggests the presence of an upper threshold that glia density cannot exceed.

Finally, we wanted to study possible correlations between glia and neuron type densities (Figures 6H,I). While hierarchically close regions show the same clustering as previously, there does not seem to be any well-defined correlation between the density of glia and that of excitatory and inhibitory neurons. While this is not surprising, it confirms that neither of these neuron types seems to have a requirement for higher number of surrounding glia.

Additional correlations between cell type densities and ratios in the brain can be found in Appendix section 1.3 in Supplementary Material.

### Validation against additional literature and automated counting

Validating the generated densities and numbers is not trivial as only 138 literature values were available, with only 38 regions having 2 or more values reporting the density of the same cell type, without counting the numbers reported by Murakami et al. (2018) and Kim et al. (2017). There are also multiple intrinsic deviations that are difficult to take into account, such as inter-subject variability and subject age differences. Additionally, the cell counting methods used in literature can all vary, as well as the atlas used to delimitate the specific regions studied. As a result, numbers varied by a median of 1.8-fold and a mean of 4.1-fold in cases where more than one study reported on the same region or area, with up to 13.1-fold for certain regions.

**Figure 7:**
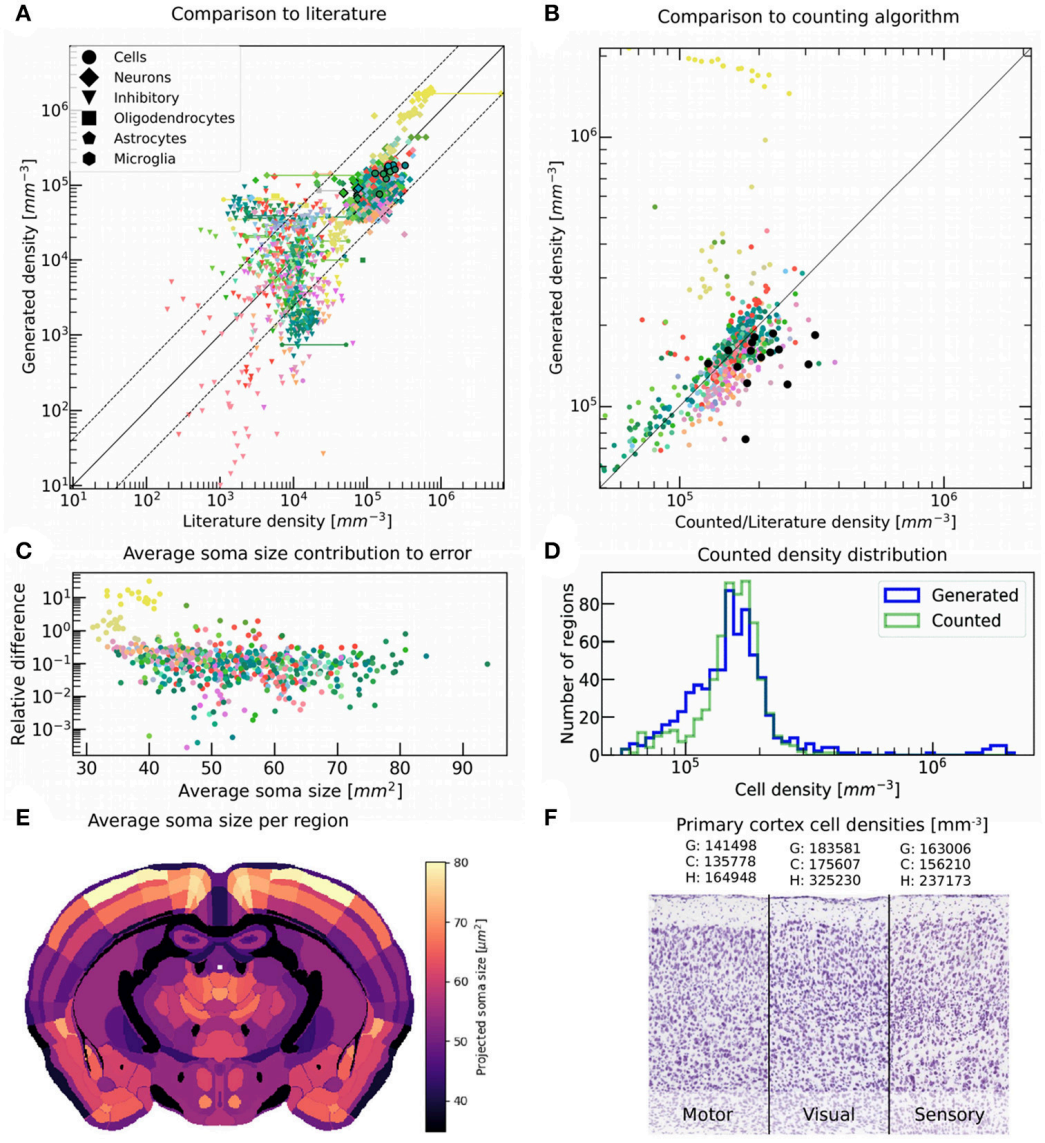
Validation of generated cell, glia and neuron densities against literature data and counted numbers. **(A)** Comparison between generated densities of different cell types and literature values reporting the same quantity, and that were not used during the generation process. When multiple literature sources are available for the exact same region, they are both shown as a data point and are linked together. The color encodes the brain regions according to the AMBA, while the shapes of the points encode for cell types. The middle line delimits equal quantities, while the dashed line shows the average deviation of 2.7-fold between literature values reporting on the same region. Data points outlined in black originate from Herculano-Houzel et al. (2013). This comparison includes Murakami et al. (2018) and Kim et al. (2017) who provided the majority of numbers for neurons and inhibitory neurons. **(B)** Comparison between cell densities generated, and counted by the automatic point-detection algorithm for every region of the brain. Colors are as in **(A)**. The middle line delimits equal quantities. The data points for cell density that are available in literature and are also shown in **(A)**, are represented as black dots for comparison. **(C)** Contribution of average soma size to the relative error between generated and counted cell densities, for each region. The relative error was defined as the difference between generated and counted densities, divided by the counted densities. Overall, most regions seem to exhibit a relative error of 10% between the two methods, with the lowest deviations reaching 0.1% and the highest being around 1,000% mostly for cerebellar regions. The lack of correlation shows that the density approximation was not systematically undermined by the deviations in the observed soma size. **(D)** Histograms of regions in the brain according to cell density, for generated (blue) and counted (green) numbers. The main difference lies in the long-tail which is only present for the generated numbers, as the counting algorithm does not work properly for highly dense regions. **(E)** Average soma size per region, approximated by the automatic counting algorithm, and shown in coronal view. **(F)** Different cortical regions as seen on the original Nissl stained slice from AIBS, with their average cell densities obtained from our generation algorithm (G), our automatic point-detection algorithm (C), and from literature (Herculano-Houzel et al., 2013) (H).

Overall most generated numbers were reasonably well-aligned to their experimental counterparts (Figure 7A). Some deviations could be observed however and seemed to be highest for cerebellar regions, whereas isocortical numbers were predicted more reliably. A reason for this is about half of the numbers for isocortical regions originating from Herculano-Houzel et al. (2013) (Figure 7A, black outline) and thus being sampled using the same technique, thus leading to a greater overall consistency. In contrast, other sources focused on individual regions, each being measured with a different technique. Additionally, both the experimental delimitation of regions and the cell counting were done manually in most cases, leading to a further chasm between reported numbers. Even though we converted all reported regions to their approximate equivalent in the Allen Brain Atlas, this might have resulted in additional deviations. In fact, the main motivation behind comparing cell densities rather than absolute numbers between our model and the literature was to minimize the impact caused by differences in region size. A better overview of Figure 7A for each cell type is shown in Appendix section 1.4 in Supplementary Material.

To obtain some estimate of the ground-truth, we used the regional cell numbers obtained by our automatic point-detection algorithm for low density areas (Figure 7B). This served as a consistency check, as it used the same Nissl stained microscopy images that the volumetric cell density data set used by our generation workflow was based on (Figure 1C). As expected, the counted density always stayed below a threshold of around 3·10^5^ mm^−3^, as the algorithm failed to distinguish overlapping cell bodies (Figure 1C). Accordingly, the deviation from generated numbers was the largest for highly dense regions such as the cerebellum (Figure 7B). Other regions showed a good agreement between the generated and counted numbers, being even closer than generated and literature values compared earlier. This finding was further contrasted by the fact that almost all cell density numbers from literature originated from Herculano-Houzel et al. (2013), which was considered to be one of the more consistent sources. The histogram of cell densities for all regions showed a good match between generated and counted numbers (Figure 7D), besides the long-tail of the distribution which was expectedly not present for the counted numbers.

According to literature, the primary visual cortex (V1) has a cell density around 50% higher than other cortical regions (Herculano-Houzel et al., 2013) (Figure 7F). Our method however only provided a value around 20% higher. A similar number was found by the automatic counting algorithm, applied on the microscopy images of the same subject. This seems to suggest that some of the deviations originate either from inter-subject variability or from the microscopy imaging itself, and are difficult to account for.

Our spatial density estimation assumed a constant cell body size throughout the brain, which is never the case in reality. In an effort to compensate for this effect, we extended our point-detection algorithm to further estimate the average soma size as well in each region (Figure 7E). This was done by fitting the image of each detected soma by a 2-dimensional circular Heaviside step function, until the best matching radius was found. This method was not very accurate for highly dense regions however, as the detection of overlapping cells was limited there (Figure 1C). We found no systematic correlation between the relative error between generated and counted numbers, and the average soma size in the same region (Figure 7C). As a result, we did not use these results to apply a density correction factor to compensate for varying soma sizes.

### Web-based interface

**Figure 8:**
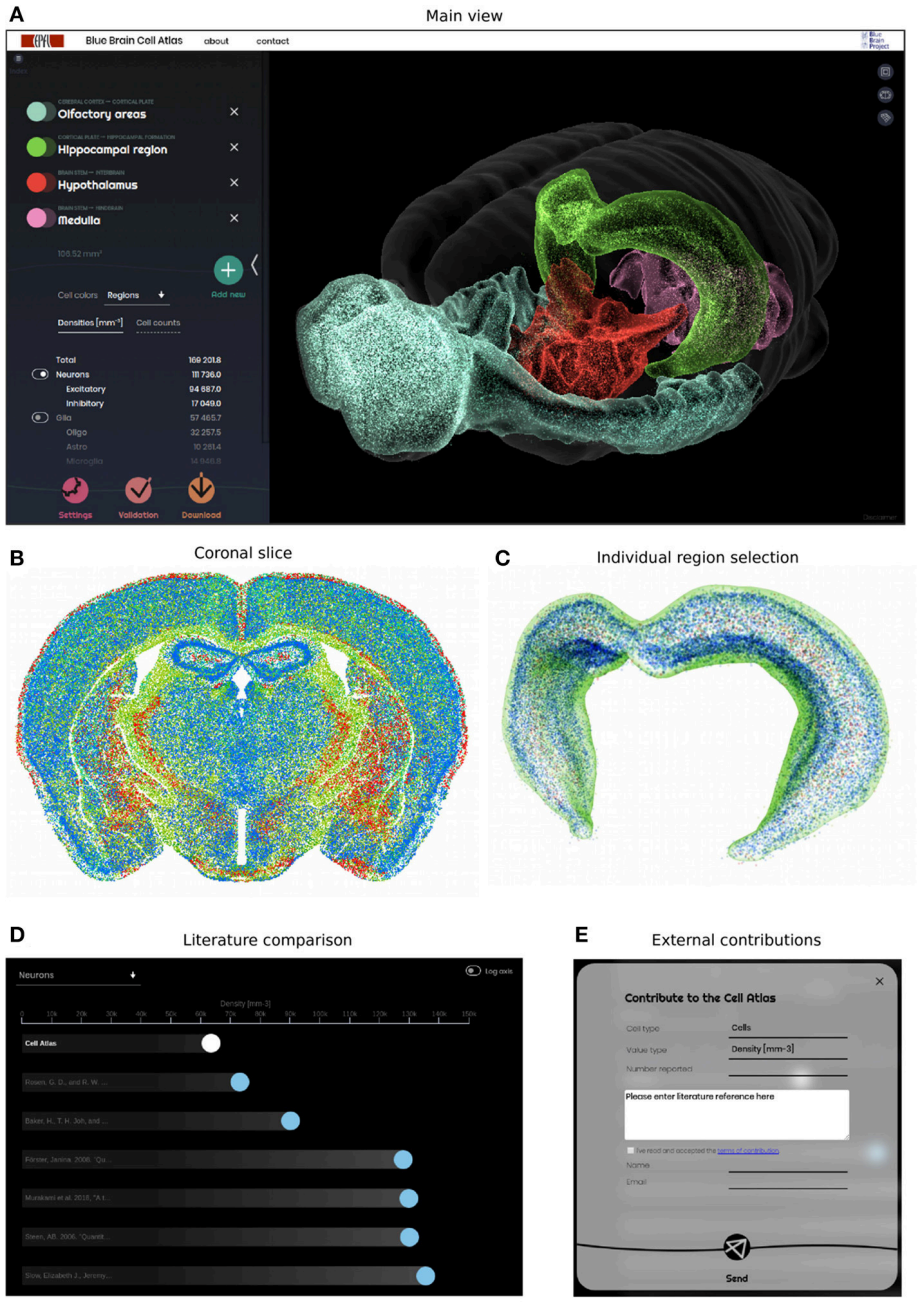
Web-based Cell Atlas providing an overview of all neurons and glia in the brain. **(A)** Global view of the cell atlas, allowing the selection of multiple regions and their display in 3D, color-coded according to the Allen Brain Atlas (Dong, 2008). The website displays 1% of all cells as small dots, at their reconstructed position in space. The cell counts and densities are shown on the left panel, along with the selected regions. The positions and densities of all cells in the brain can be downloaded. **(B)** Coronal view of the brain, showing 100% of cells in a 25 μm–thick virtual slice. The colors reflect cell type in this case, but can be changed with a dynamic interface. **(C)** Regions can be selected and studied individually, as shown here for the Hippocampus. **(D)** Validation panel, comparing the Cell Atlas densities for each selected region and cell type, with their literature counterparts. Each dot represents a literature number on a linear or logarithmic axis, and can be selected to access the underlying literature reference. White dot represents our reconstructed number for the selected region. **(E)** Contribution panel, allowing external users to enter region and cell type specific numbers for further validation. This encourages a collaborative effort to accumulate further literature numbers, and converge toward a ground-truth.

The 3D cell atlas (bbp.epfl.ch/nexus/cell-atlas) has been designed to provide a global overview of the brain cell composition, while also allowing the user to study specific regions in detail (Figures 8A–C). Most importantly, it shows the confidence and progress of the reconstruction effort (Figure 8D), and allows researchers to contribute their own numbers to the validation effort (Figure 8E).

## Discussion

We provide for the first time estimates of the number of neurons, glia, and their subtypes in all known regions of the brain. These were achieved using a data-driven approach that allows for convergence toward reference values found in literature. This resource allows researchers to query the cellular composition of the entire brain, but also of very specific areas. Analyzing the data provides ratios and correlations between cell types in every brain region, yielding deeper insight into the cellular organization of the mouse brain. As our method for obtaining and differentiating cells is relatively simple, it can also be easily extended to integrate additional genetic markers, or more generally whole-brain datasets for any cell type. The same approach and model can therefore be used to develop a catalog of cell-types in every brain region at even greater level of detail based on molecular, genetic, and electrophysiological data. Finally, the exact cell positions are also provided by the workflow, allowing the building of computer models of the whole mouse brain, or of brain systems composed of single or multiple regions.

### Cell densities and correlation

The number of glia and cells (neurons and glia) are proportional for low cell densities and breaks down as cell densities surpass 2.5·10^5^ mm^−3^. This was further confirmed by the constant offset present between cell and neuron densities, indicating an upper ceiling for glial density. Neuron densities could thus be an order of magnitude higher than glia densities in certain regions. Overall glia density also appeared to be much more uniform throughout the brain than other cells, which was also suggested by its much narrower distribution across the regions. Indeed, while neuron and cell densities followed a long-tailed distribution even on a logarithmic scale, glia densities did not. There was no particular correlation between glia and specific neuron types, indicating no preference for glia toward one or the other.

The cerebellum was consistently over-represented in terms of excitatory neuron density, due to the presence of granule cells. It was however similar to other regions for other cell types, suggesting that a high granule cell density would not significantly alter the structural organization of other cell types.

### Validation against literature

We reviewed a large array of available literature values for cell, neuron and glia numbers in the mouse brain. A large disagreement between multiple numbers reported for the same brain region could however be observed. These varied by a median of 1.8-fold and a mean of 4.1-fold, up to 13.1-fold in some cases. This makes it difficult to construct a grand truth for cell densities from individual literature sources alone.

We were however able to compare our numbers to their literature equivalent. We found an overall decent agreement for most cell types, mostly within the deviation that different literature sources presented to themselves.

Some of the approximations made during our workflow contributed to these deviations. First, the mathematical function *f(V)* used to obtain density values from voxel intensities assumed constant cell sizes and large voxel depth. The second was the alignment of genetic marker datasets, which despite being made as accurate as possible was still relying on manual input and a finite number of fiducial points. Furthermore, some artifacts originating from the acquisition itself were visible on the stained images and could not be corrected.

Other deviations most likely resulted from the inaccuracy in regional delimitation, as well as from the heterogeneity of counting techniques employed by the different research groups. These problems were however unavoidable when retrieving data from multiple sources. Inter-subject variability and difference in the age of the subject were other innate sources of error that could only be avoided if all density measurements were performed on a single animal. Finally, the main problem was the difficulty of dissociating these variables from each other, as their contribution to the deviations was impossible to systematically account for.

### Validation against automated counting

To get another approximation of the ground-truth, we also run an automated cell counting algorithm on the original Nissl dataset. While this algorithm was applied to the 2-dimensional slices, it yielded 3-dimensional positions for all cells by using the coronal position of each slice in the brain. These in turn resulted in region-specific total counts that could directly be compared to those obtained using the density-based generation algorithm. This would also ensure the lack of errors due to inter-subject variability and age difference, as well as due to differences in measurement techniques.

Numbers matched well in the regions where the counting algorithm worked, and unsurprisingly less so in high density regions. Both generated and counted numbers exhibited a similar spread throughout the brain, besides for the long-tail of the distribution which was not present in the case of the counting algorithm. There was overall a better agreement between generated and counted numbers, than between generated and literature numbers. This was not surprising, as both the counting algorithm and the volumetric density used in by our method were based on the same Nissl stained microscopy images, thereby excluding inter-subject variability from the deviations. The average soma sizes obtained by the counting algorithm could not be used to correct the error caused by the constant soma size assumption, as there was no systematic correlation between them.

We could not find a significantly higher density in the visual cortex than in other cortical areas, despite predictions by literature numbers. However, both the results from the counting algorithm and a direct qualitative observation of the original Nissl stained slice showed similar densities between main isocortical areas. The error might therefore be due to inter-subject variability between species, which is present despite the uniform use of wild-type mice.

Using point-detection techniques on higher density microscopy images might alleviate some of the problems encountered here. It would however also create new challenges in terms of differentiating cells into multiple types, as it cannot be applied to multiple subjects without causing mismatch between cell positions.

### Future work

Our approach is a way to consolidate current knowledge about the brain from available data. Any deficiencies of the model highlight scarcity or issues in the underlying data and assumptions. Therefore, an important benefit of our approach is that it helps to guide future research and suggests new experiments, because we can estimate from the validations which new data would improve the model most. Thus, the benefit of this large-scale model derives as much from what it cannot explain as from what it can. One therefore has to keep in mind that the model itself is only a reflection of the underlying datasets.

Future iterations will improve upon the modules delineated here. We will employ the latest AIBS atlas instead of generating a new aligned version. We will combine all genetic markers available from the AIBS, and attribute a list of genes to every cell of our model. We will then apply all known exclusion and inclusion rules to the labeled cells to obtain additional cell types. We will quantify the expected variation in cell numbers within the target strain, thereby allowing individual instances to be created. In the future, we also seek to integrate connectivity data into our model. These will include tracer injection experiments from the AIBS, and from other initiatives such as the Brain Architecture Project (mouse.brainarchitecture.org), the Mouse Connectome Project (mouseconnectome.org) or the MouseLight Project (janelia.org/project-team/mouselight). The fact that most of these initiatives use the reference space from the AIBS would facilitate this task. Finally, the cell positions generated in this approach will also be used as the basis for building a whole-brain network employing whole neuron morphologies (Markram et al., 2015).

## Author contributions

CE developed the algorithm and created the web interface. DK collected the literature data for validation and identified key genetic markers. All authors wrote the text.

### Conflict of interest statement

The authors declare that the research was conducted in the absence of any commercial or financial relationships that could be construed as a potential conflict of interest.
