# Corrigendum: A Cell Atlas for the Mouse Brain

**DOI:** 10.3389/fninf.2019.00007

**Published:** 2019-02-19

**Authors:** Csaba Erö, Marc-Oliver Gewaltig, Daniel Keller, Henry Markram

**Affiliations:** Blue Brain Project, École Polytechnique Fédérale de Lausanne (EPFL), Lausanne, Switzerland

**Keywords:** cell numbers, neurons, glia, mouse brain, Allen brain atlas

In the original article, there was an error in the funding section.

A correction has been made to the **Funding** section and now states:

“This work was funded by the EPFL Blue Brain Project Fund and the ETH Board Funding to the Blue Brain Project. The work has been performed as an in-kind contribution to the European Union Seventh Framework Programme (FP7/2007-2013) under grant agreement no. 604102 (Human Brain Project) and the European Union's Horizon 2020 Framework Programme for Research and Innovation under Grant Agreement No. 720270 (Human Brain Project SGA1).”

The authors apologize for this error and state that this does not change the scientific conclusions of the article in any way. The original article has been updated.

