# Supplementary material for: A Cell Atlas for the Mouse Brain
Source: Front Neuroinform. 2018 Nov 28;12:84. doi: 10.3389/fninf.2018.00084 (PMC6280067; doi:10.3389/fninf.2018.00084)
Supplement: Supplementary file 3 [file Data_Sheet_3.docx]

# Appendix

## Automated non-rigid alignment

In order to apply a non-rigid automated alignment procedure to the volumetric dataset, we first convolved the voxels of the Nissl volume with a 3-dimensional Gaussian kernel (σ= 2 voxels) to prevent the alignment algorithm from getting caught in local minima. Each 2-dimensional section was also individually convolved with a Gaussian kernel of the same size. The slice planes of the filtered reference atlas were then passed through an automated non-rigid alignment algorithm (Kroon, 2008), which generated a grid of translation vectors needed to align the images. These were then applied to both the original as well as to the reference annotated images. This alignment step not only improved the offset between adjacent slices, but also partially corrected the spatial warping in the images created during acquisition (**Fig. 2b**). The result is a realigned 3-dimensional atlas with the same voxel resolution as the reference atlas but with an improved structural alignment. This new atlas was used in all subsequent stages of the analysis.

## Cell overlap compensation function

The AIBS created the volumetric reference atlas by averaging the grayscale values of all pixels of the Nissl stained microscopy images into lower resolution voxels. As a first approximation, this value can be interpreted as the density of cell bodies inside each voxel. However, this approach systematically underestimates the cell density: due to the non-negligible thickness of the slice (25μm), some cell bodies may occlude others, preventing them from contributing to the observed cell density observed in the 2-dimensional microscopy images (**Fig. 2d**). To alleviate this effect, we derived a simple transfer function *D=f(V)* and applied it to all voxel values.

Let *s* be the surface of a cell as projected on a 2D plane and S_tot_ the total surface of the plane contributing to a voxel. Further let V_n_ be the observed fraction of 2D space occupied when the voxel contains *n* cell bodies. Then, for up to two cell bodies in a voxel we have:

$$V_{0}=0$$

$$V_{1}=\frac{s}{S_{\mathrm{tot}}}$$

$$V_{2}=V_{1}+\left( 1-V_{1} \right)\cdot\frac{s}{S_{\mathrm{tot}}}$$

From this, we can derive an iterative equation for the addition of each new cell body to the voxel volume. Assuming that the size of a cell body is smaller than the thickness of the slice, the mean amount of free space is given by the total area minus the already occupied area, obtaining the term (1-V_1_). We can generalize this equation to obtain an expression for the area occupied by *n* cell bodies V_n+1_, given V_n_:

$$V_{n+1}=V_{n}+(1-V_{n})\cdot\frac{s}{S_{\mathrm{tot}}}$$

$$V_{n+1}=V_{n} \cdot(1-\frac{s}{S_{\mathrm{tot}}}) + \frac{s}{S_{\mathrm{tot}}}$$

$$V_{n+1}= a\cdot V_{n}+b$$

This expression can be rewritten as arithmetic-geometric sequence, using the substitutions a=1-s/S_tot_ and b=s/S_tot_, obtaining the general form of:

$$V_{n}=a^{n} \cdot\left( V_{0}-\frac{b}{1-a} \right)+\frac{b}{1-a}$$

$$V_{n}=\frac{b}{1-a} \cdot\left( 1-\left( 1-\frac{s}{S_{\mathrm{tot}}} \right)^{n} \right)$$

$$V_{n}=1-\left( 1-\frac{s}{S_{\mathrm{tot}}} \right)^{n}$$

From this, we obtain the estimated number of cells *n* in a voxel as:

$$n=\frac{ln(1-V_{n})}{ln(1-s/S_{\mathrm{tot}})}$$

As the average surface occupied by a cell body is unknown and the cell body density is proportional to the cell count, we can write the final density as:

$$f\left( V \right)\propto n= -ln({1-V}_{n})\cdot A$$

(**Fig. 2d**) where *A* is a positive constant assumed uniform throughout space. Fortunately, the density *D* is normalized later during the cell creation and specification procedures using literature constraints and therefore *A* does not need to be known explicitly. We can check the validity of *f(V)* by estimating *A*. To do this, we applied f on the voxel data set and used the total cell number for the whole brain (Herculano-Houzel et al. 2011):

$$A=N_{\mathrm{tot}}/\sum_{i} -ln(1-V_{n}^{i})=16.47$$

This allowed us to obtain an approximation for the average projected cell surface *s* and thus its radius.

$${s=S}_{\mathrm{tot}}\cdot(1-exp(-1/A))$$

This derivation also allowed us to approximate the average cell radius as 3.41μm, and to use this number to validate the transfer function, *f(V)*. By randomly placing spheres of this radius into a voxel of 25μm^3^ volume while using spatial exclusion, we could measure the density of the voxel and compare it to the actual number of cells inside (**Fig. 2d**). The analytical function matched the numerical results reasonably well, being only slightly deficient for very high densities, probably because the calculation of the approximation function did not take spatial exclusion in account.

## Additional cell type correlations


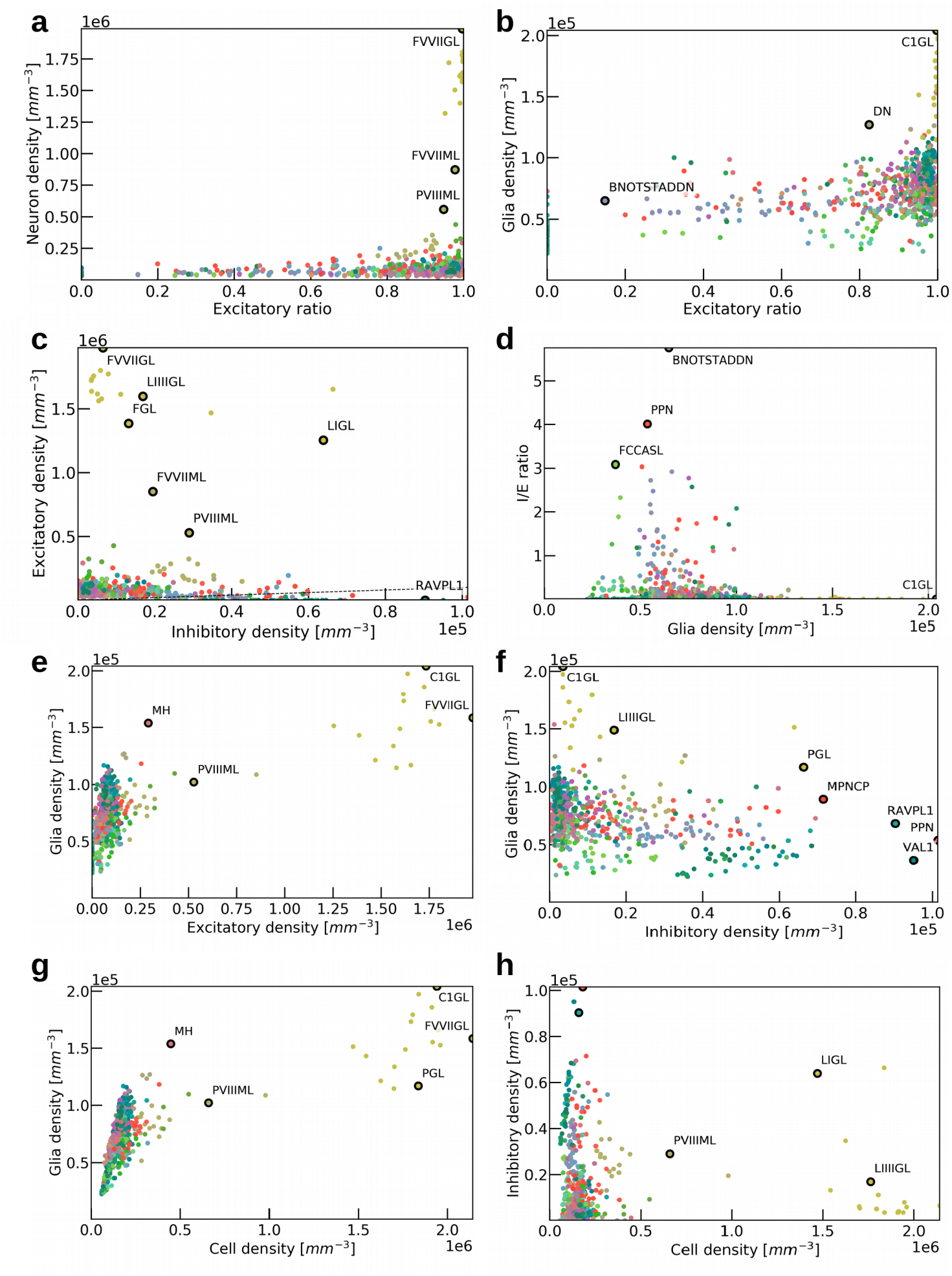


***Figure S1*** *Correlation between cell type densities or ratios of different brain regions. Each dot represents a region of the brain and is color-coded according to the Allen Brain Atlas annotation. Quantities displayed are the inter-dependence of neural and glial density and the excitatory ratio (****ab****), excitatory and inhibitory densities (****c****), and E/I ratios and glia density (****d****). The dependence between glia density and excitatory and inhibitory densities is further shown (****ef****), as well as between glia and inhibitory densities, and the overall cell density (****gh****). Abbreviations are: Retrosplenial area, ventral part, layer 1 (RAVPL1), Pyramus VIII, molecular layer (PVIIIML), Posterodorsal preoptic nucleus (PPN), Lingula I, granular layer (LIGL), Lobule III, granular layer (LIIIIGL), arbor vitae (V), dorsal limb (L), Folium-tuber vermis VII, granular layer (FVVIIGL), Folium-tuber vermis VII, molecular layer (FVVIIML), Flocculus, granular layer (FGL), Bed nuclei of the stria terminalis, anterior division, dorsomedial nucleus (BNOTSTADDN).*

## Validating regional cell densities


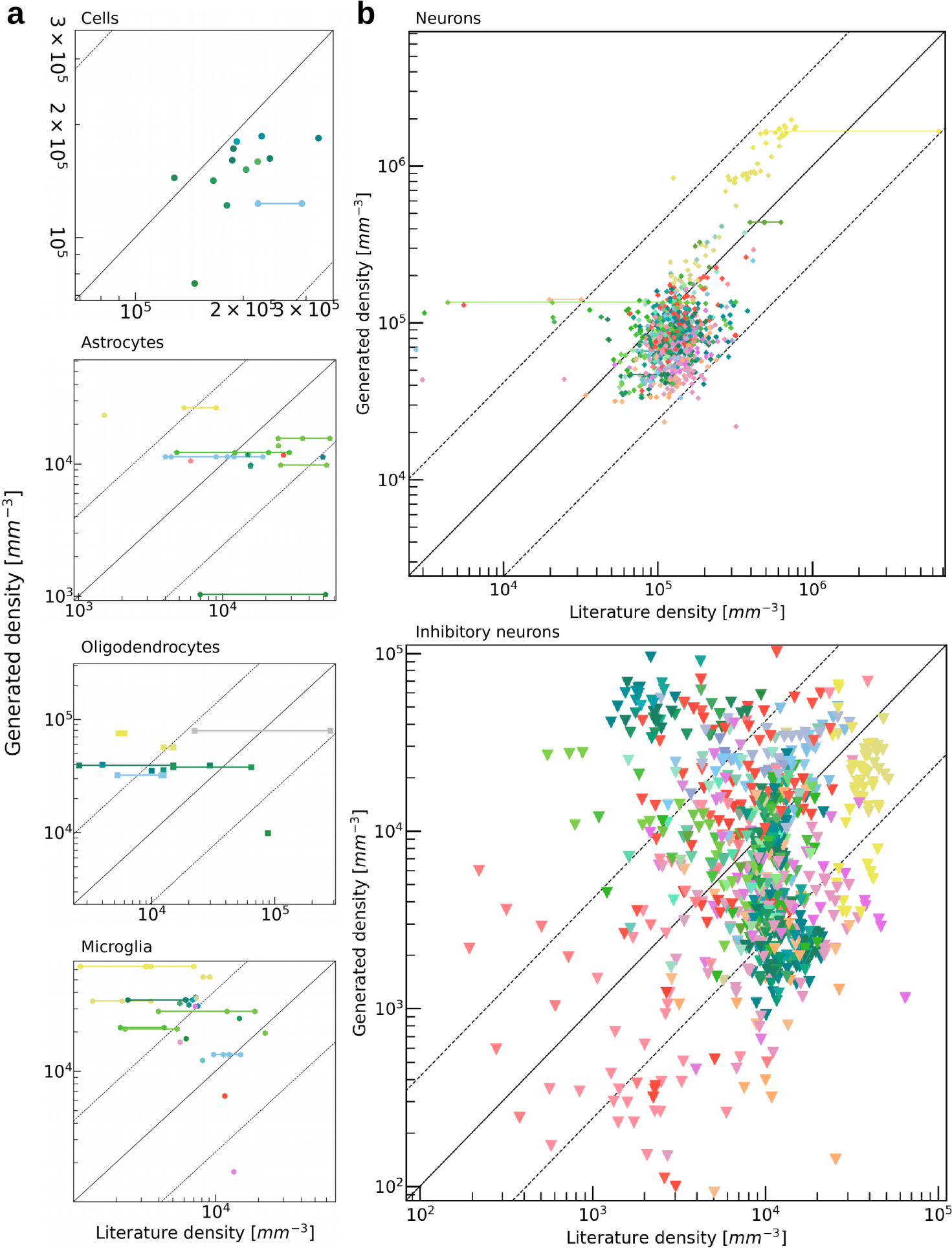


***Figure S2*** *Validation of generated cell, glia and neuron densities against literature data. Comparison between generated densities of different cell types and literature values reporting the same quantity, and that were not used during the generation process. Multiple literature sources available for the same region are shown as linked data points.
(****a****) Comparison of numbers available for cell, astrocyte, oligodendrocyte and microglia densities, against their literature counterparts.
(****b****) Neuronal and inhibitory neuron density comparison against literature. These numbers were much more numerous, as they were systematically reported for all regions by Murakami, et al. (2018) and Kim, et al. (2017).*
